# Short- and Long-Term Transcriptomic Responses of Escherichia coli to Biocides: a Systems Analysis

**DOI:** 10.1128/AEM.00708-20

**Published:** 2020-07-02

**Authors:** Beatriz Merchel Piovesan Pereira, Xiaokang Wang, Ilias Tagkopoulos

**Affiliations:** a Microbiology Graduate Group, University of California, Davis, California, USA; b Genome Center, University of California, Davis, California, USA; c Biomedical Engineering Graduate Group, University of California, Davis, California, USA; d Department of Computer Science, University of California, Davis, California, USA; University of Tartu

**Keywords:** antiseptics, biocides, disinfectants, microbial resistance, stress response, transcriptomics

## Abstract

The mechanisms of the bacterial response to biocides are poorly understood, despite their broad application. To identify the genetic basis and pathways implicated in the biocide stress response, we exposed Escherichia coli populations to 10 ubiquitous biocides. By comparing the transcriptional responses between a short-term exposure (30 min) and a long-term exposure (8 to 12 h) to biocide stress, we established the common gene and pathway clusters that are implicated in general and biocide-specific stress responses. Our analysis revealed a temporal choreography, starting from the upregulation of chaperones to the subsequent repression of motility and chemotaxis pathways and the induction of an anaerobic pool of enzymes and biofilm regulators. A systematic analysis of the transcriptional data identified a *zur*-regulated gene cluster to be highly active in the stress response against sodium hypochlorite and peracetic acid, presenting a link between the biocide stress response and zinc homeostasis. Susceptibility assays with knockout mutants further validated our findings and provide clear targets for downstream investigation of the implicated mechanisms of action.

**IMPORTANCE** Antiseptics and disinfectant products are of great importance to control and eliminate pathogens, especially in settings such as hospitals and the food industry. Such products are widely distributed and frequently poorly regulated. Occasional outbreaks have been associated with microbes resistant to such compounds, and researchers have indicated potential cross-resistance with antibiotics. Despite that, there are many gaps in knowledge about the bacterial stress response and the mechanisms of microbial resistance to antiseptics and disinfectants. We investigated the stress response of the bacterium Escherichia coli to 10 common disinfectant and antiseptic chemicals to shed light on the potential mechanisms of tolerance to such compounds.

## Abstract (precis)

Antiseptics and disinfectant products are of great importance to control and eliminate pathogens, especially in settings such as hospitals and the food industry. Such products are widely distributed and frequently poorly regulated. Occasional outbreaks have been associated with microbes resistant to such compounds, and researchers have indicated potential cross-resistance with antibiotics. Despite that, there are many gaps in knowledge about the bacterial stress response and the mechanisms of microbial resistance to antiseptics and disinfectants. We investigated the stress response of the bacterium Escherichia coli to 10 common disinfectant and antiseptic chemicals to shed light on the potential mechanisms of tolerance to such compounds.

## INTRODUCTION

Biocides, by the definition of the European Commission, include antiseptics, disinfectants, and preservatives. They are intended to control, eliminate, or reduce the number of undesired organisms, similar to antibiotics, which are used to eradicate infections in humans and animals (1). Several researchers have suggested the possibility of cross-resistance between biocides and antibiotics or other biocides (1–5). In contrast to the microbial mechanistic response to antibiotics, which has been extensively studied, there is a lack of understanding regarding the microbial response to biocides (6, 7), and the Food and Drug Administration (FDA) has recently expressed the need to collect more data related to biocide cross-resistance (8).

Research on biocide resistance is limited, partially due to a general belief that, unlike antibiotics, their multitarget action in the cell does not select for resistance to a specific target (9). In contrast, exposure to a subinhibitory concentration of triclosan, a broad-spectrum bisphenol biocide, was shown to select for Escherichia coli mutants with mutations in the *fabI* gene (10). *fabI* is homologous to genes found in other species, such as Staphylococcus aureus, and is also the target of therapeutic drugs against tuberculosis (11). Triclosan had been added to various household products for at least a decade, until very recently, when the FDA took action to remove this compound from most products since neither the safety nor the efficacy of triclosan has been shown (12). As the scientific community adds to our knowledge regarding the safety of biocide utilization, regulatory agencies become able to provide consumers and manufacturers with updated guidelines for the adequate use of these compounds.

Bacterial adaptation to biocides, which may include changes in gene expression as well as selection for mutants, may emerge for various reasons. Those include the irregular use of biocidal products, a gradient distribution around corners and difficult-to-reach areas, and improper disposal in the environment (7, 13). Aside from triclosan’s selection of *fabI* mutants (9, 10), multidrug efflux pumps have been one of the few well-studied mechanisms implicated in adaptation to biocides (14). Additional mechanisms of resistance and tolerance to biocides, such as changes in biofilm formation (15) and activation of *soxRS* and *oxyR* by oxidative agents, have also been proposed (16). Overall modification of the membrane composition has been reported and indirectly associated with resistant and cross-resistant phenotypes (17–19). Additionally, several *Pseudomonas* spp. are capable of degrading biocides such as quaternary ammonium compounds (7, 20). In contrast, the bacterial response to other biocides, such as povidone-iodine (POV) and glutaraldehyde (GLUTA), has not been studied, even though strains isolated from the environment were tolerant to such chemicals (21).

Transcriptomic data for microbes exposed to biocides can provide valuable information regarding the bacterial responses to subinhibitory concentrations of these compounds. Few studies have explored the genome-wide molecular responses of Escherichia coli and other bacteria to biocides. The E. coli expression profile following exposure to hydrogen peroxide has been studied by various groups, including ours (22, 23). A few researchers have explored the transcriptomics of different bacteria exposed to sodium hypochlorite (SOD) (24–26), ethanol (ETOH) (27, 28), povidone-iodine (25), benzalkonium chloride (BENZ) (24, 25), peracetic acid (PERA) (24, 29), and chlorhexidine (XID) (30, 31). Research on biocides has focused on a few compounds at a time under a range of experimental conditions with different protocols, strains, and media across research groups, making it difficult to compare data across these dimensions.

**FIG 1:**
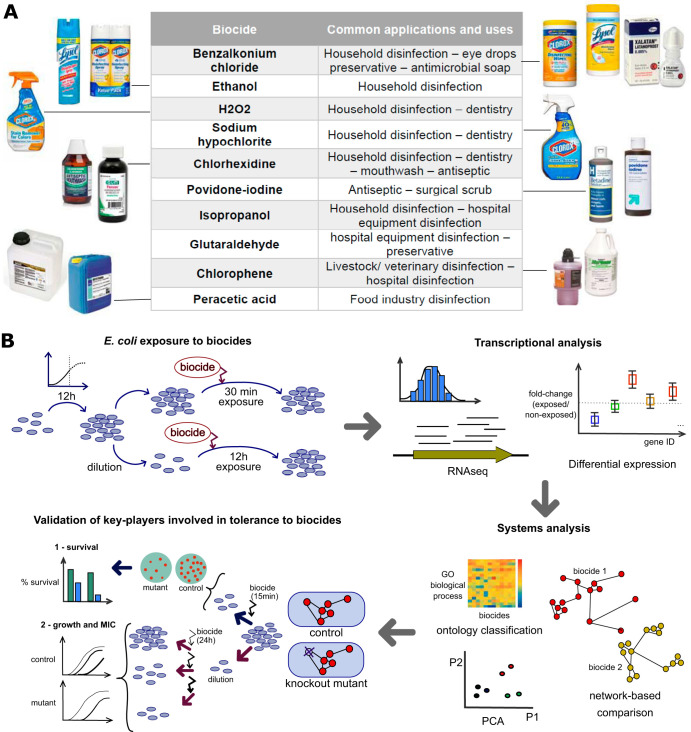
Systems analysis of E. coli response to biocide stress. (A) Ten biocides frequently used in residential and commercial applications were selected. (B) Overview of the experimental and computational setting. E. coli MG1655 cells were grown for 12 h in minimal medium (M9 medium) with 0.4% glucose and then exposed for either a short term (30 min) or a long term (8 to 12 h) to the biocides benzalkonium chloride, chlorophene, chlorhexidine, hydrogen peroxide, glutaraldehyde, ethanol, isopropanol, peracetic acid, sodium hypochlorite, or povidone-iodine. Samples were taken for genome-wide transcriptomics profiling, determination of differentially expressed genes (DEGs), and Gene Ontology (GO) analysis. Biocide susceptibility was assayed on selected knockout mutants through the use of growth curves and survival assays.

To bridge this gap, we first created a cohesive transcriptomics data set of E. coli MG1655 responses to subinhibitory concentrations of 10 commonly used biocides under otherwise identical conditions (Fig. 1A). We then identified both common and biocide-specific stress responses to each biocidal compound and performed differential expression and ontological analysis to elucidate the key players and their role in the bacterial response to specific biocides. We further validated the effects of those candidate genes by assessing the fitness effects of their knockouts through the use of survival assays and growth curves (Fig. 1B).

## RESULTS

### The general, biocide-agnostic, stress response dominated gene expression.

**FIG 2:**
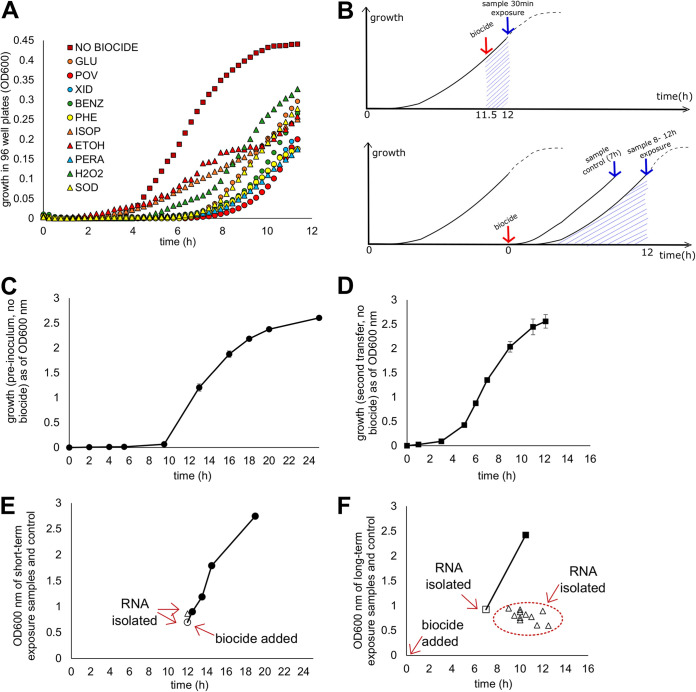
Growth and sample collection protocol. (A) The growth curves for E. coli MG1655 in 96-well plates with and without the presence of each biocide at the concentration picked for transcriptomics assays. (B) Growth and sample collection protocol for samples exposed to biocide for the short (top) and long (bottom) term. (C) Typical growth of E. coli MG1655 in assay tubes without biocide, starting from a frozen culture. (D) Typical growth of E. coli MG1655 in assay tubes without biocide, starting from dilution of the preinoculum depicted in panel C. (E) Actual OD_600_ values for samples with a short-term exposure and the control at the time of collection (open circle, control; open triangle, samples with biocide exposure). Closed circles represent the trajectory of growth of the control after sample collection. (F) Actual OD_600_ values for samples with long-term exposure and the control at the time of collection (open square, control; open triangles, samples with biocide exposure). The closed square represents the trajectory of growth of the control after sample collection.

**FIG 3:**
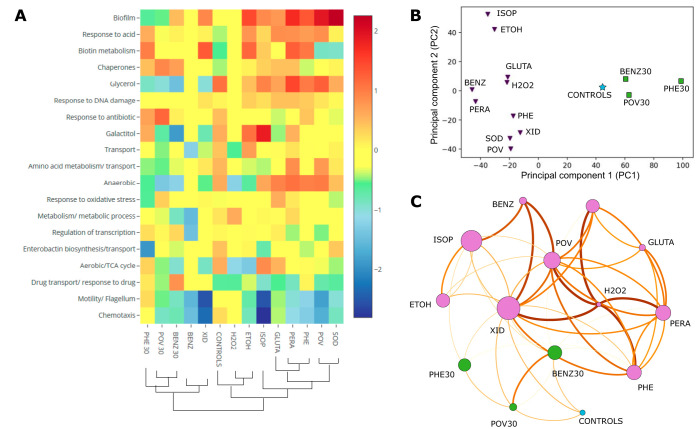
Transcriptomic response of E. coli to 10 commonly used biocides. (A) DEGs (*q* < 0.05) from RNA-seq were organized into clusters based on GO biological process terms. The average between the log_2_ fold change in expression of DEGs belonging to the same category was calculated, and the biological processes were organized in descending order of differential regulation. (B) Principal-component analysis (PCA) for the DEGs identified (triplicates). Squares and triangles correspond to the samples with short- and long-term exposures, respectively. The star represents the result for the comparison between the two sets of controls. (C) Network connections between the biocide conditions. The node size depends on the number of DEGs for the specified biocide condition, and the connections between nodes (edges) are based on shared DEGs. A higher edge thickness indicates a higher number of shared DEGs compared to the total number of DEGs for the condition. Only the top three connections per condition are shown. BENZ, benzalkonium chloride; BENZ30, benzalkonium chloride exposure for 30 min; ETOH, ethanol; GLUTA, glutaraldehyde; H_2_O_2_, hydrogen peroxide; ISOP, isopropanol; PERA, peracetic acid; PHE, chlorophene; PHE30, chlorophene exposure for 30 min; POV, povidone-iodine; POV30, povidone-iodine exposure for 30 min; SOD, sodium hypochlorite; XID, chlorhexidine.

We evaluated the gene expression for Escherichia coli after continued short-term (30-min) and long-term (8-h to 12-h) exposure to 10 biocides (Fig. 1 and Table 1; see also Table S1 in the supplemental material and Materials and Methods for details). Transcriptomic samples were collected at mid-exponential growth (Fig. 2; see Materials and Methods for more information). We observed variation in the number of differentially expressed genes (DEGs) across conditions (205 to 396 genes in the short-term exposure versus 14 to 672 genes in the long-term exposure; Table S2). The short-term response to biocides was characterized by the upregulation of chaperones (14 DEGs; average log_2_ fold change [FC] in expression, 0.70) and response to drugs or antibiotics (22 DEGs; average log_2_ FC, 0.67). In comparison, the long-term response included the upregulation of biofilm formation (7 DEGs; average log_2_ FC, 1.13), the downregulation of chemotaxis (23 DEGs; average log_2_ FC, –1.27), and the downregulation of motility and flagellum assembly (45 DEGs; average log_2_ FC, –1.27) (Fig. 3A; Table 2; Fig. S1A).

**TABLE 1:** Biocides utilized in this work^*a*^

Biocide	Abbreviation	Concn (transcriptomics)	Neutralizer(s) (survival assay)	Group	Mode of action
Benzalkonium chloride	BENZ	3.63 mg/liter	Lecithin (0.5%) and Tween 80 (1%)	Cationic agent (QAC)	Membrane damage
Chlorhexidine	XID	1.48 μM	Lecithin (0.5%) and Tween 80 1%)	Cationic biguanide	Membrane damage
Chlorophene	PHE	0.25 μM	Lecithin (0.5%) and Tween 80 (1%)	Halogenated phenolic	Inhibits membrane-bound proteins
Glutaraldehyde	GLUTA	29 μM	Sodium bisulfite (1%)	Aldehyde	Protein denaturation and cross-linkage
Hydrogen peroxide	H_2_O_2_	272 μM	NA	Peroxygen	Oxidative
Ethanol	ETOH	2.8% (vol/vol)	Dilution only	Alcohol	Membrane damage and protein denaturation
Isopropanol	ISOP	2.7% (vol/vol)	Dilution only	Alcohol	Membrane damage and protein denaturation
Peracetic acid	PERA	9 μM	Sodium thiosulfate (1%) and Tween 80 (1%)	Peroxygen	Oxidative
Povidone-iodine	POV	12.5 μg/ml	Sodium thiosulfate (1%)	Halogen	Interacts with thiol groups on proteins
Sodium hypochlorite	SOD	3.64 μM	Sodium thiosulfate (1%)	Chlorine	Oxidative

a The concentrations to which E. coli cells were exposed before samples were taken for transcriptomic assays are shown. The concentrations of biocides used for transcriptomics were determined to give, on average, 50% growth inhibition (according to the OD_600_) at 12 h compared to the growth of a control not exposed to a biocide. NA, not applicable (the assay was not performed); QAC, quaternary ammonium compound.

**TABLE 2:** Biological processes that were the most affected for each condition^*a*^

Condition	Most affected biological process (score)	
	Upregulated	Downregulated
BENZ	NA	Motility/flagellum (−1.56)
XID	Biotin metabolism (1.31)	Motility/flagellum (−2.52)
PHE	Biofilm (1.33)	Chemotaxis (−1.28)
GLUTA	Anaerobic growth (0.95)	Drug transport/response (−0.57)
H_2_O_2_	Metabolism (0.62)	Aerobic growth (−1.15)
ETOH	Biofilm (1.46)	Aerobic growth (−1.33)
ISOP	Galactitol (1.86)	Chemotaxis (−2.80)
PERA	Biofilm (1.72)	Chemotaxis (−1.09)
POV	Biofilm (1.92)	Motility/flagellum (−1.81)
SOD	Biofilm (2.26)	Motility/flagellum (−0.98)
Long term	Biofilm (1.13)	Motility/flagellum (−1.27)
Long term	Glycerol (0.55)	Chemotaxis (−1.27)
BENZ30	Drug transport/response (0.82)	Glycerol (−1.44)
PHE30	Biotin metabolism (1.00)	Enterobactin (−1.92)
POV30	Response to antibiotic (1.19)	Anaerobic growth (−1.34)
Short term	Chaperones (0.70)	Glycerol (−1.11)
Short term	Response to antibiotic (0.67)	Anaerobic growth (−1.05)

a The score corresponds to the average of the log_2_ fold change in expression for all the DEGs belonging to the corresponding pathway/biological process. The top up- and downregulated processes were also calculated for the average of all the conditions belonging to either short- or long-term exposure. NA, not applicable.

**FIG 4:**
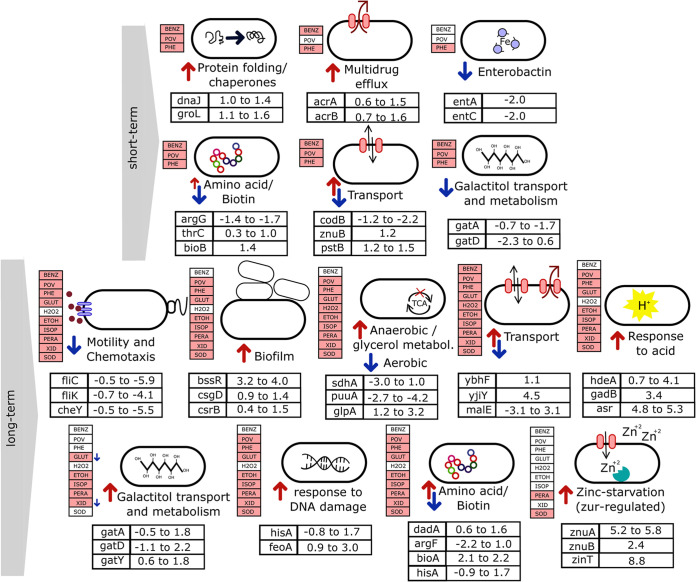
E. coli responses to short- and long-term biocide exposure. Upregulated processes (red arrows) and downregulated processes (blue arrows) after subinhibitory short-term (3 biocides) or long-term (10 biocides) biocide exposure. Biocides were colored when the biological process was affected (at least one DEG belonged to that category) after exposure to the biocide. Representative DEGs for each biological process are shown along with their log_2_ fold change in expression over all biocides. Detailed information with the complete list of DEGs can be found in Table S1 in the supplemental material. metabol., metabolism.

Principal-component analysis (PCA) of the DEG signatures revealed grouping of the samples into distinct groups that correlated with the time of exposure (short or long). While for the two alcohols, ethanol and isopropanol (ISOP), the various samples clustered together, as expected, the samples corresponding to short-term exposure formed a cluster separate from those exposed to the same biocide but for a longer duration (Fig. 3B; Fig. S1B). Among the most informative genes determined by sparse PCA, there were genes related to motility, respiration, the acid stress response, biofilm formation, and transport (Fig. S1C and D), all of which are processes controlled by genes that were differentially expressed distinctly between samples with short-term exposure and samples with long-term exposure. The network analysis recapitulated the clustering results together with common DEG associations (Fig. 3C), and an overview of the temporal dynamics is provided in Fig. 4.

### Protein folding was an early response to biocide stress.

The short-term exposure (30 min) of E. coli to biocides (BENZ for 30 min [BENZ30], chlorophene [PHE] for 30 min [PHE30], and POV for 30 min [POV30]) resulted in high levels of overexpression of several chaperones and cochaperonins, such as *dnaK*, *dnaJ*, *groL*, *groS*, *htpG*, *hscA*, *cpxP*, and *clpB*. Interestingly, this was not observed for cells exposed to the same biocides for more extended periods (Fig. 3A; Table S1). Exposure to povidone-iodine for 30 min resulted in the overexpression of *ibpB* (a molecular chaperone), which was among the genes for which the highest log_2_ FC (>6.1) was observed for the entire transcriptome data set (Table S3). Hence, the stress response to misfolded proteins appears to be an early response of the cell to stress, replaced later by other types of protective adaptations, such as biofilm formation. The exception was the chaperone gene *spy*, which was highly upregulated by long-term exposure to isopropanol. This chaperone has previously been shown to be highly upregulated in E. coli exposed to other alcohols (68).

### Long-term exposure to biocides induced biofilm formation and shut down motility.

Long-term exposure (8 to 12 h) of E. coli to biocide stress resulted in the downregulation of genes related to motility and chemotaxis (Fig. 3A). Overall, this was a strong response for all biocides, except for glutaraldehyde (GLUTA) and H_2_O_2_ (Fig. 4). Downregulation was more expressive for cells exposed to chlorhexidine (XID) and isopropanol (ISOP) than for the cells exposed to the remaining conditions. For both biocides, the most downregulated gene was related to motility (Table S3). The promotion of biofilm formation occurs through the inhibition of motility (32), which is consistent with our results. Transcriptomic data revealed a strong indication of the induction of biofilm formation by long-term biocide exposure, even though different regulators were expressed in each group of biocides, such as *bssR*, *csgD*, and *csrB.* Our results indicate that although different biocide treatments may trigger the same outcome response (biofilm formation) by E. coli, diverse pathways and regulators are preferentially activated, depending on the agent utilized.

### Long-term exposure to biocides rewired respiration pathways, induced anaerobiosis, and shut down the TCA cycle.

A significant number of genes related to the tricarboxylic acid (TCA) cycle were downregulated after the long-term exposure of E. coli to biocides (Fig. 3A; Table 3; Table S1), such as *sdhBC* and *gltA*. Accordingly, several genes appeared to be differentially regulated in the direction of an anaerobic state of the cells: *cydAB*, *ompW*, *glpABC*, and *nrdD* were upregulated, while *katE*, *puuA*, and *acnA* were downregulated (Table 3). Taken together, the evidence indicates that an anaerobic state of E. coli cells was induced by long-term biocide exposure.

**TABLE 3:** Common genes involved in the biological processes affected by long-term biocide exposure^*a*^

Process	Gene(s)	Description (reference)	Regulation	Condition(s)
Motility	*fli* series	Related to flagellum	Down	BENZ, ETOH, GLUTA, ISOP, PERA, PHE, POV, SOD, XID
	*flg* series	Related to flagellum	Down	BENZ, ETOH, ISOP, PERA, PHE, POV, SOD, XID
	*cheY*, *cheZ*	Chemotaxis/motility	Down	BENZ, ETOH, ISOP, PERA, PHE, POV, SOD, XID
	*motA*, *motB*	Motility protein	Down	BENZ, ETOH, ISOP, PERA, PHE, POV, SOD, XID
Biofilm	*bssR*	Regulator of biofilm formation	Up	PERA, PHE, POV, SOD
	*csgD*	Transcriptional regulator	Up	GLUTA, ISOP, PHE, XID
	*csrB*	Regulatory RNA	Up	ETOH, ISOP, PERA, PHE, XID
Aerobic/anaerobic growth	*sdhB*, *sdhC*, *sdhA*	Succinate dehydrogenase; part of the TCA cycle	Down	ETOH, H_2_O_2_, PERA, PHE, POV, SOD, XID
	*gltA*	Citrate synthase; part of the TCA cycle	Down	ETOH, PERA, PHE, POV, SOD, XID
	*cyA*, *cyB*	Cytochrome components; induced under oxygen limitation conditions (58)	Up	POV, PERA, XID, SOD, PHE, ISOP, ETOH, GLUTA
	*ompW*	Outer membrane protein; upregulated during the transition from the aerobic to the anaerobic state (59)	Up	PERA, PHE, POV, SOD
	*glpABC*	Glycerol-3-phosphate dehydrogenase; functional under anaerobic conditions	Up	PERA, PHE, POV, SOD
	*nrdD*	DNA synthesis and repair; functional under anaerobic conditions (60)	Up	GLUTA, PHE, SOD, POV, PERA
	*katE*	Response to oxidative stress; upregulated in the aerobic state compared to its regulation during anaerobiosis (61)	Down	ETOH
	*puuA*	Putrescine utilization pathway; upregulated during the transition from the anaerobic to the aerobic state and downregulated in the absence of oxygen (62)	Down	ETOH, ISOP, SOD, XID PERA, PHE, POV
	*acnA*	Citric acid and glyoxylate cycles; repressed during anaerobiosis (63)	Down	ETOH, SOD
Acid stress	*hdeA*, *hdeB*	Chaperone	Up	POV, XID, PHE, PERA, SOD, GLUTA
	*gadB*, *gadC*	Glutamate decarboxylase	Up	XID, PHE, GLUTA
	*asr*	Acid shock protein	Up	ETOH, ISOP
DNA damage	*hisA*	Isomerase	Up	PERA, PHE, POV, SOD, XID
	*feoA*, *feoB*	Iron transport proteins	Up	ETOH, PERA, PHE, POV, SOD, XID
	*deoABC*	Thymidine phosphorylase	Up	ETOH, PERA, PHE, POV, SOD, XID
Stress response	*osmC*	Has peroxidase activity and accumulates under some other stress conditions, such as osmotic stress (64)	Up	XID
	*uspGF*	General stress factors with increased expression under several stress conditions (65)	Up	PERA, PHE
	*gpmM*	Role in oxidative stress response (66)	Up	PERA, POV
	*wrbA*	Unclear physiological functions in the cell	Up	PERA, PHE, POV, XID
	*zinT*	Protein that binds cadmium and zinc; was proposed to be a general stress factor with an unknown mechanism and a possible interaction role with ABC transporters (67)	Up	SOD
Response to antibiotics	*acrA*	Multidrug efflux protein	Up	PHE, PHE30, BENZ30
	*ybjG*	Phosphatase	Up	BENZ30
	*phoU*	Phosphate signaling protein	Up	BENZ30, POV30, ISOP, ETOH
	*ybhG*	Role in drug resistance (34)	Up	PHE, PHE30
	*sseA*	3-Mercaptopyruvate sulfurtransferase	Up	XID, ISOP

a A summary of the genes up- or downregulated after long-term exposure to biocides is shown. The complete list with the respective log_2_ fold change in expression and *P* values can be found in Table S1 in the supplemental material. When the reference is not explicit, the gene function was obtained from the EcoCyc database (55).

### The acid stress response was ubiquitously present after long-term exposure to biocides and varied in its regulation.

The strong upregulation of genes associated with the response to acid functional category was observed across biocides after long-term exposure (Fig. 3A; Table S1). The genes *hdeA* and *hdeB* were upregulated upon exposure to some of the biocides, while *gadB* and *gadC* were upregulated upon exposure to others (Table 3). Additionally, *asr* was upregulated in both alcohols (Table 3). For cells exposed to povidone-iodine, *hdeA* was the most upregulated gene (Table S3). Similar to what was observed for biofilm regulators, different genes associated with the response to acid were preferentially overexpressed after exposure to each biocide. Still, the common outcome (in which genes involved in the functional category response to acid were upregulated) was consistently observed across treatments. None of the genes mentioned above was upregulated after short-term exposure to biocides (BENZ30, PHE30, and POV30), suggesting a role of the response to acid preferentially during the late stress response.

### Biocide exposure upregulated the response to stress, DNA damage, and antibiotic response.

Several genes associated with the cellular response to DNA damage (33) were differentially expressed after biocide exposure. Among those, some were highly upregulated (log_2_ FC, >1), such as *hisA*, the *feoAB* operon, and the *deoABC* operon (Table 3).

Genes associated with the response to stress were also highly upregulated (log_2_ FC, >1) after the long-term exposure of E. coli to biocides, such as *osmC*, *uspG*, *uspF*, *gpmM*, *wrbA*, and *zinT* (Table 3). In particular, the *zinT* gene was highly upregulated after treatment with sodium hypochlorite, exhibiting the highest log_2_ FC (8.7) of the entire data set.

Cross-resistance between antimicrobials is a matter of increasing concern, and biocide exposure can potentially select for bacteria with increased tolerance to biocides and antibiotics (2–5). Among the genes that were differentially expressed after biocide exposure and that belong to the functional category response to antibiotic were *acrA*, encoding a multidrug efflux protein; *ybjG*, encoding a membrane protein; *phoU*, encoding a negative regulator; *ybhG*, encoding a putative membrane protein; and *sseA*, encoding the sulfurtransferase enzyme (Table 3).

### Multidrug efflux pumps were not a general biocide response mechanism but specific to chlorophene, benzalkonium chloride, and chlorhexidine.

Multidrug efflux has been one of the most discussed processes related to biocide tolerance, mainly due to its importance for cross-resistance to antibiotics. Contrary to expectations, we found that this mechanism was upregulated only in the case of benzalkonium chloride, chlorhexidine, and chlorophene. Both the *ybhF* gene, encoding the multidrug ABC transporter, and the *ybhG* gene, encoding a membrane protein, which are suggested to play a role in the efflux of antibiotics, such as cefoperazone and chloramphenicol (34), were upregulated after short- and long-term exposure to PHE. The multidrug efflux protein gene *mdtK* was upregulated after exposure to both PHE and XID (Table S1).

**FIG 5:**
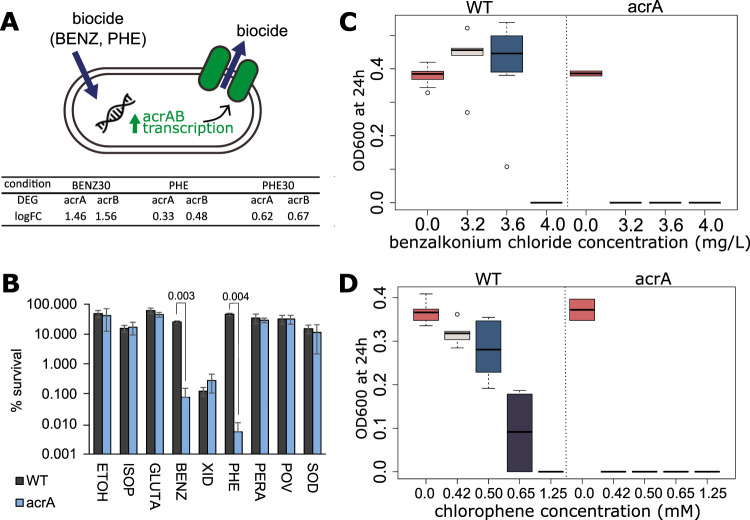
Biocide susceptibility for the E. coli mutant with a knockout of the multidrug efflux protein gene *acrA*. (A) The genes for the multidrug efflux system AcrAB were upregulated after exposure to benzalkonium chloride and chlorophene. (B) Percent survival of the mutant *acrA* and parental strain (WT) exposed to the indicated biocides for 15 min. The concentrations were as follows: ETOH, 15% (vol/vol); ISOP, 11% (vol/vol); GLUTA, 125 μM; BENZ, 12.8 mg/liter; XID, 42 μM; PHE, 0.5 mM; PERA, 18 μM; POV, 33 μg/ml; SOD, 80 μM. Error bars represent the standard errors for biological duplicates, and the *P* values for significance are indicated at the top of the bars. (C and D) Growth at 24 h in the presence of the indicated concentration of the biocide in 96-well plates. (C) Benzalkonium chloride; (D) chlorophene. The definitions of the abbreviations are as follows: BENZ, benzalkonium chloride; XID, chlorhexidine gluconate; PHE, chlorophene; ETOH, ethanol; ISOP, isopropanol; GLUTA, glutaraldehyde; SOD, sodium hypochlorite; POV, povidone-iodine; SOD, sodium hypochlorite; WT, wild-type E. coli BW25113.

Besides those, AcrAB is one of the best-described and best-characterized pumps. The proteins contribute to antimicrobial resistance by decreasing the internal cell concentration of a wide range of compounds, such as antibiotics, detergents, and dyes (14). Also, *acrB* has been shown to play a role in enterobactin export (35). The multidrug efflux genes *acrAB* were upregulated after exposure to BENZ for 30 min (BENZ30) and after both short- and long-term exposure to chlorophene (PHE30 and PHE, respectively). The upregulation of *acrAB* may protect cells against harmful concentrations of such biocides by decreasing their level inside the cell (Fig. 5A). We evaluated the effect of exposure to BENZ, PHE, and seven additional biocides for an *acrA* knockout mutant. We observed that the absence of the efflux pump affected E. coli survival, which was specifically found for BENZ and PHE (Fig. 5B). We confirmed the increased susceptibility of the *acrA* mutant to the biocides BENZ and PHE with 96-well plate growth assays (Fig. 5C and D).

### Zinc starvation genes played a role in E. coli survival after exposure to sodium hypochlorite and peracetic acid.

**FIG 6:**
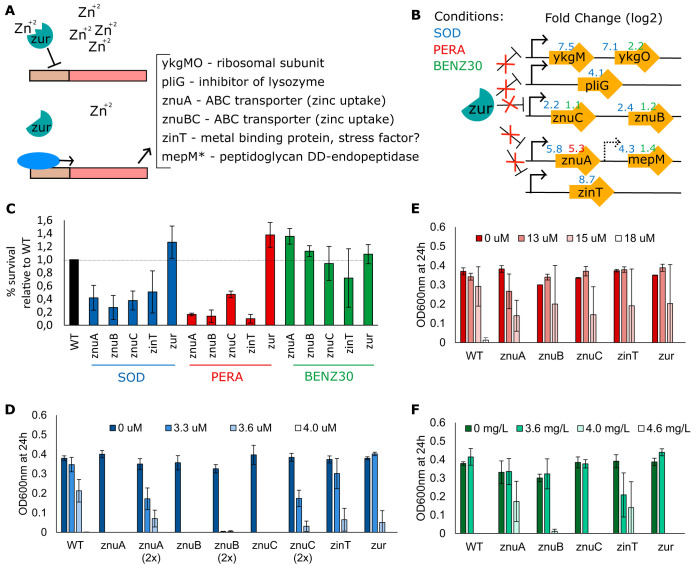
Exposure to biocides induces a zinc starvation response. (A) Zinc starvation disengages the transcriptional regulator *zur* from DNA binding (37), resulting in overexpression of *zur*-regulated genes (37–40). (B) Long-term exposure to sodium hypochlorite (SOD) and peracetic acid (PERA) and a short-term 30-min exposure to benzalkonium chloride (BENZ30) resulted in the overexpression of *zur*-regulated genes. (C) Percent survival relative to that of the wild type (E. coli BW25113) for the indicated mutants and E. coli MG1655 in SOD at 49 μM, PERA at 18 μM, and BENZ at 13 mg/liter. (D to F) Growth at 24 h in the presence of the indicated concentration of the biocide in 96-well plates. (D) Sodium hypochlorite; (E) peracetic acid; (F) benzalkonium chloride. Error bars represent standard errors for biological replicates.

Zinc homeostasis in bacterial cells is maintained by uptake and export systems tightly regulated by their regulators, which include the Zur repressors and members of the Fur protein family of iron regulators. The metalloprotein Zur has a high affinity for zinc (36) and is proposed to regulate the zinc transporter genes *znuABC* (37), *zinT* (previously *yodA*) (38), *pliG* (39), and the putative ribosomal protein genes *ykgMO* (40) by binding to the DNA when zinc is abundant and repressing expression of the genes (Fig. 6A). We observed the overexpression of genes proposed to be regulated by *zur* after E. coli exposure to sodium hypochlorite (SOD), peracetic acid (PERA), and benzalkonium chloride (BENZ30) (Fig. 6B), even though no difference in expression of *zur* itself was detected. Expression levels were considerably higher for SOD and PERA, with log_2_ FC values being above 5 (Fig. 6B; Table S3).

We further investigated the survival of E. coli knockout mutants for *znuABC*, *zinT*, and *zur* after exposure to SOD, PERA, or BENZ for 15 min, followed by neutralization and plating into agar medium. The relative survival of the *znuA*, *znuB*, *znuC*, and *zinT* mutants was significantly lower than that of the wild-type strain after exposure to both SOD and PERA (Fig. 6C). On the other hand, a mutant for the repressor *zur* showed an increased ability to survive exposure to the biocides, which could have been a direct result of the derepression of the genes controlled by *zur*. Interestingly, we did not observe such an effect for mutants exposed to BENZ: no significant difference was observed between the mutants and the parent strain, except for the *znuAB* mutants, which were less sensitive to the biocide than the parent strain (Fig. 6C). We also assessed the growth of the mutants for 24 h in a plate reader in the presence of a range of concentrations of each biocide (Fig. 6D to F). Unlike the survival assay, this evaluation of growth did not include neutralization of the biocide and was an indication of growth inhibition (and not necessarily killing) by the biocides. All mutants showed increased sensitivity to SOD compared to the parent strain. However, the sensitivity of the *znuABC* mutant was considerably higher (Fig. 6D). Since the growth of the *znuA*, *znuB*, and *znuC* mutants was slower than that of the wild-type strain in minimal medium without biocide, we further assessed growth with an initial concentration of cells twice as high as that for the parent strain to minimize the effect of growth defects for evaluation of biocide susceptibility. The strains were still more susceptible to the biocide than the parent strain, even with a higher initial cell concentration (Fig. 6D). Differences between the mutants and the parent strain were not noticeable after exposure to PERA (Fig. 6E), and higher fluctuations between replicates indicated that the concentration of 15 μM was close to the limit for total growth inhibition for the cell concentration utilized in the assay. Growth in the presence of BENZ was similar to that of the wild type for most mutant strains except the *znuA* and *zinT* mutants (Fig. 6F).

## DISCUSSION

Genome-wide transcriptional profiling of the biocide response usually entails experiments in single biocides, at single time points, and under different conditions, factors which complicate comparisons and the delineation of general versus specific responses to biocide exposure (22–31).

Even though it is widely known that stress conditions generate a temporally structured gene expression response in bacterial cells (41), most studies related to biocides are limited to observations at a single time point, limiting our understanding of the bacterial response to such chemicals. Here, our inclusion of samples at early and late exposure times allowed us to observe a temporal choreography of responses and differences between short- and long-term exposure to biocides. One of these was the upregulation of chaperones during the short-term exposure to biocides, indicating that the response to misfolded proteins is exclusively an early stress response. Accordingly, the expression of amino acid-related genes was markedly distinct between cells exposed short and long term to the same biocide, which emphasizes distinct protein production requirements with treatment for each length of time.

Concomitantly, the long-term bacterial response was directed toward an anaerobic state of the cell, the downregulation of motility and chemotaxis, and the overexpression of biofilm regulators. Recently, it was demonstrated that E. coli cells growing in an anaerobic state have a higher mutation rate (42), which, combined with the selective pressure of an environment with subinhibitory concentrations of biocides, may aid with the emergence and maintenance of antimicrobial-resistant strains.

The late response of the downregulation of motility could be a strategy to save energy, as the production of flagellum requires a considerable expenditure of energy that can be invested in other strategies for protection against stress, such as biofilm formation (32). It is important to highlight that cells grouped in biofilm structures have increased tolerance to biocides (43), and the consistent induction of a biofilm-forming state by bacteria exposed to subinhibitory concentrations of biocides represents a concern for the elimination of pathogens in hospitals and food production settings.

The induction of both biofilm regulators and acid stress-related genes occurred across most biocides after long-term exposure. However, in both cases, the pathway of choice was distinct between groups of biocides. Such an observation indicates that even though the long-term exposure to biocides may result in a similar output phenotype, E. coli utilizes various mechanisms of stress response at the gene expression level tailored to each compound encountered. Whether this mechanistic radiation is because of physical and chemical cross dependencies that internalize the correlation structure of the environmental setting that E. coli has evolved in remains to be determined.

The possibility of selection of bacterial strains cross-resistant to antibiotics once they have been exposed to subinhibitory concentrations of biocides and the occurrence of cross-resistance between biocides have been studied before (2–5, 44). Several genes implicated in increased tolerance to antibiotics were differentially expressed after exposure to biocides, including the genes for multidrug efflux pumps, such as the pump encoded by *acrB*, which we also correlated to decreased susceptibility to the biocides benzalkonium chloride and chlorophene.

The transcriptomics for E. coli exposed to specific biocides also allowed us to identify a group of DEGs regulated by *zur*, the negative regulator of the zinc starvation response, which was associated with biocide susceptibility. Bacterial mutants for the zinc transport systems encoded by *znuABC* and *zupT* were previously shown to be more sensitive to hydrogen peroxide than the wild type and attenuated during infection of mouse models, indicating a competitive advantage of zinc transport systems for infection (45). The expression of *zur*-regulated genes was also simultaneous with the overexpression of biofilm regulators and the downregulation of motility. Knockout mutants for *znuABC* have impaired motility (45), and mutants for *zinT* and *ykgM* have reduced biofilm formation (46). Overexpression of zinc transporters by prolonged exposure to a subinhibitory concentration of biocides may also contribute to biofilm formation and increased fitness in subsequent host infection.

Finally, aside from its intellectual merit on bacterial physiology, dissecting the bacterial response to biocide stress and understanding the potential mechanisms of tolerance and resistance are crucial when it comes to informing future policy and guidelines on biocide use.

## MATERIALS AND METHODS

### Biocides.

Benzalkonium chloride (MP Biomedicals), hydrogen peroxide (Macron), peracetic acid (Sigma-Aldrich), sodium hypochlorite (Sigma-Aldrich), glutaraldehyde (Amresco), chlorhexidine (Aldrich), chlorhexidine gluconate (Spectrum), and povidone-iodine (Sigma) stock solutions were prepared by dilution in sterile, demineralized water. Chlorophene (Aldrich) stock solution was prepared by dilution in ethanol (Sigma-Aldrich). To refer to each biocide, we use the abbreviations provided in Table 1. All solutions, as well as ethanol (Sigma-Aldrich) and isopropanol (Spectrum), were sterilized with 0.22-μm-pore-size filters and kept in the dark at 8°C. Working solutions were prepared daily by further dilutions in sterile, demineralized water.

### Strain and culture conditions.

All bacterial samples used in this work were collected for RNA isolation at mid-exponential growth (Fig. 2). Escherichia coli MG1655 was obtained from frozen (−80°C) stock tubes and grown in minimal medium (M9 medium with 0.4% [wt/vol] glucose) for 12 h (mid-exponential growth) at 37°C (preinoculum). Next, the cells were diluted 1:100 into fresh M9 medium with 0.4% glucose containing 1 of the 10 biocides (Fig. 1 and 2). The biocides tested were benzalkonium chloride, isopropanol, ethanol, hydrogen peroxide, peracetic acid, sodium hypochlorite, glutaraldehyde, chlorophene, chlorhexidine, or povidone-iodine (Table 1). The subinhibitory concentrations utilized for each biocide were determined previously to provide, on average, 50% growth inhibition (according to the optical density at 600 nm [OD_600_]) after 12 h of exposure compared to the growth of a control without biocide when grown in a plate reader (Fig. 2A). Groth rates were not affected by most biocides except for ethanol and isopropanol. Instead, the cells showed an extended lag phase when growing in the presence of the biocides (Fig. 2A).The final biocide concentrations were as follows: benzalkonium chloride, 3.63 mg/liter; isopropanol, 2.7% (vol/vol); ethanol, 2.8% (vol/vol); hydrogen peroxide, 272 μM; peracetic acid, 9 μM; sodium hypochlorite, 3.64 μM; glutaraldehyde, 29 μM; chlorophene, 0.25 mM; chlorhexidine, 1.48 μM; and povidone-iodine, 12.5 μg/ml. The final concentration of ethanol for the experiments with chlorophene exposure was 0.5% (vol/vol). For sample collection, growth was stopped after 8 to 12 h, which corresponded to mid-exponential growth, when the OD_600_ reached values ranging from 0.6 to 0.95 (Fig. 2F). Tubes without biocide served as a control. For the control, growth was also stopped at the mid-exponential stage, at 7 h of growth. Cold 5% (vol/vol) phenol-ethanol was added (1.5 ml per 3 ml of sample) to each tube. The cells were pelleted by centrifugation at 4,000 rpm at 4°C for 10 min and stored at −80°C for up to 2 weeks. The preinoculum cells were also prepared as described above and stored for further analysis.

Additionally, preinoculum cells were also exposed to benzalkonium chloride, chlorophene, or povidone-iodine at the concentrations mentioned above, without further cell dilution, for 30 min (Fig. 1B and 2E) and stored as described above. Chlorophene was selected for the short-term-exposure evaluation due to the lack of information in the scientific literature regarding the microbial response to this biocide. Povidone-iodine and benzalkonium chloride were included for the short-term-exposure evaluation given the opposite claims regarding the development of bacterial resistance to these compounds. Adaptation to increasing concentrations of benzalkonium chloride as well as acquired cross-resistance to additional antimicrobials after prolonged exposure to the biocide has been reported (3, 44). Povidone-iodine, on the other hand, was claimed to have superior antimicrobial activity and has repeatedly been reported to be an antiseptic that does not select for resistance in bacterial strains (47, 48). All experiments were performed in triplicate.

### RNA extraction and transcriptomics.

Total RNA was recovered from pelleted cells with an RNeasy minikit (Qiagen) and on-column DNase digestion (Qiagen). rRNA was removed with a capture oligonucleotide mix (MICROBExpress; Ambion). The total RNA concentration (3 μg) was previously optimized by our group to increase the ribosomal depletion yield for the kit (average amount of rRNA removed, above 99%). RNA cleanup was performed with a NucleoSpin RNA cleanup kit (Macherey-Nagel). For library preparation, a Kapa Stranded transcriptome sequencing (RNA-seq) library preparation kit for Illumina platforms (Kapa Biosystems) was used according to the manufacturer’s instructions. An extra step of size selection was performed using Agencourt AMPure XP magnetic beads (Beckman Coulter). The DNA concentration for each sample library was determined with a Qbit (v.2.0) fluorometer (Invitrogen). The DNA concentration of the final pooled library was determined with a Bioanalyzer DNA high-sensitivity assay (Agilent; DNA Technologies Core, University of California at Davis [UCDavis]). Sequencing was performed with a HiSeq 3000/4000 SR50 sequencer (DNA Technologies Core, UCDavis).

### Transcriptomics data analysis.

Adapters and low-quality reads were removed from the raw reads by use of the Trimmomatic trimmer (49), followed by alignment to the reference files for E. coli MG1655 (50) by use of the Bowtie2 program (51). Then, the bam files generated by Bowtie2 were fed into the FeatureCounts program (52), yielding the counts for each gene in each replicate. We examined the proximity between replicates for different biocides by constructing a multidimensional plot over the counts for all the genes (53). Finally, differentially expressed genes (DEGs) were identified using the edgeR (53) and Deseq-2 (54) programs, with a false discovery rate of 0.05 being used as the threshold for calling differential expression. The common DEGs across these two approaches were reported as final DEGs. For gene expression analysis, the averages of the log_2_ fold change (log_2_ FC) in expression obtained by the two methods were used.

The expression data generated from RNA-seq were comprehensively analyzed for gene functionality, location in the cell, pathways, relationships between genes, and additional information with the EcoCyc (55) and STRING databases. The Cytoscape (v.3.4.0) program (56) with the STRING application (default settings of a confidence score cutoff of 0.4 and a maximum number of interactors of 0 were used) was used to build and analyze the gene networks for DEGs with absolute log_2_ FC values greater than 1. Each gene cluster generated by the program was manually screened for the processes or pathways present. Gene Ontology (GO) terms (biological process, molecular function, and cellular component) were captured from the EcoCyc database for all DEGs. The complete list of DEGs (*q* < 0.05) was used to build a heatmap with the biological processes affected by each biocide. All information was combined to express the processes most significantly affected by biocide exposure. Differentially expressed genes (DEGs) were classified and ranked according to the log_2_ FC. The DEGs that appeared under multiple conditions (equal to or more than three), as well as the DEGs with the highest absolute log_2_ FC values, were evaluated for the GO term biological process and included in the list of the most significant processes affected by biocide exposure.

### Biocide exposure assays.

Mutants with knockouts of selected genes (*acrA*, *znuABC*, *zinT*, *zur*) were obtained from the Keio Collection (57). All strains were verified regarding the correct position of the kanamycin insert by PCR and sequencing. The primers used are listed in Table 4. The effects of biocide exposure on the knockout mutants were tested with growth curves and survival assays. For growth curve experiments, the mutants were grown overnight in minimal medium (M9 medium) with 0.4% (wt/vol) glucose. The OD_600_ was measured and adjusted to 0.1 ± 0.05. Two microliters of the cell cultures was transferred to each well of 96-well plates containing biocides at a range of concentrations diluted in M9 medium with glucose to a total of 200 μl/well. Both the Keio Collection parent strain E. coli BW25113 and strain MG1655 were used as controls and exposed to the same concentrations of the biocides for comparison. The plates were incubated for 24 h at 37°C with agitation in a plate reader (Synergy HT; BioTek). For survival assays, overnight cultures were diluted in M9 medium-glucose and grown to exponential phase (OD_600_, 0.5 ± 0.3). The OD_600_ was adjusted to 0.19 for a constant cell concentration across experiments. Cells were challenged with the biocides for 15 min at 37°C with agitation. After the addition of the appropriate neutralizer (Table 1), cells were diluted in 0.9% saline and plated on LB agar. Percent survival was calculated for each mutant by comparison to the survival of a control not exposed to the biocide. Results were normalized by considering the survival for the parent strain E. coli BW25113 (wild type [WT]) to be 100%.

**TABLE 4:** Primers used in this work^*a*^

Primer no.	Primer name	Primer sequence
1	kan_check_knockout	CCGTGATATTGCTGAAGAGC
2	kan_check_knockout_2	GTTTCTGCGGACTGGCTTTC
3	check_acrA_knockout_fw	GTATGTACCATAGCACGACG
4	check_acrA_knockout_rv	CATGATGATAATGGCGATCAC
5	check_zur_knockout_fw	CATTACGGCAACAATAAGGG
6	check_zur_knockout_rv	AACCCGCAATGAATATCGC
7	check_zinT_knockout_fw	CTGAGAAAGCCATGCTCTCG
8	check_zinT_knockout_rv	TAGCTTGCGTTCAGTGGC
9	check_znuA_knockout_fw	CGGGCTATCTGTTGCACG
10	check_znuA_knockout_rv	CCAGCGACACATCAGAGA
11	check_znuB_knockout_fw	GGTGCTGAACAACTGGGT
12	check_znuB_knockout_rv	AGGTCGGATAAGGCGCTC
13	check_znuC_knockout_fw	TTGCACCTCCCCAGAGAG
14	check_znuC_knockout_rv	CAAACGAACCCAGCGGAC
		
15	ydcI fw	TTCTTGACGCCATCAACACTGCCG
16	ydcI rv	GCAAGGTCGTCTCTTTTTGTTGCTG
17	yccJ fw	GCTCATCACGTCGGTGAATGGG
18	yccJ rv	CCTTCTTCCCAAATCTTTTCCGCC
19	yjcZ fw	GGCACTGACGCAGATCGC
20	yjcZ rv	ACCTGCCTGCACCAGTAGG
21	znuA fw	GCGGACTTAGTCGTTTGGGTTGG
22	znuA rv	GCGTGGTCGTGATCATCATCATCG

a Primers 1 to 14 were used to check the correctness of the knockout strains from the Keio Collection (57) utilized in this work. Primers 3 to 14 were used to amplify the region expected to have the deletion according to the scheme shown in Fig. S2 in the supplemental material (primer PCR). Primers 1 and 2 (primer sequences) were used independently to verify the correct insertion of the kanamycin resistance gene and the deletion of the gene *acrA*, *zur*, *zinT*, *znuA*, *znuB*, or *znuC* in the strains. Primers 15 to 22 were used for quantitative PCR validation of the DNA sequencing data. See also Fig S3 in the supplemental material. fw, forward; rv, reverse.

### Availability of data.

The data generated or analyzed during this study are included in this published article (and in the supplemental material. RNA-seq data are deposited in the NCBI Gene Expression Omnibus (GEO) database under accession number GSE124673.

## Supplementary Material

Supplemental file 1

Supplemental file 2
