# Supplementary material for: Short- and Long-Term Transcriptomic Responses of Escherichia coli to Biocides: a Systems Analysis
Source: Appl Environ Microbiol. 2020 Jul 2;86(14):e00708-20. doi: 10.1128/AEM.00708-20 (PMC7357472; doi:10.1128/AEM.00708-20)
Supplement: Supplemental file 1 [file AEM.00708-20-s0001.pdf]

A systems analysis of *E. coli* short and long-term response to biocides

Beatriz Merchel Piovesan Pereira[1,2], Xiaokang Wang[2,3], Ilias Tagkopoulos*[2,4]

1. Microbiology Graduate Group, University of California, Davis, USA
2. Genome Center, University of California, Davis, CA, USA
3. Biomedical Engineering Graduate Group, University of California, Davis, USA
4. Department of Computer Science, University of California, Davis, USA
   *Corresponding author

SUPPLEMENTARY ONLINE MATERIAL

SUPPLEMENTARY METHODS

qPCR

Differential expression of selected genes detected by RNAseq (*ydcI, yccJ, yjcZ, and znuA*) was validated with qPCR (**Fig. S3**). For cDNA synthesis, the RevertAid (Thermo Scientific) kit was used, according to the manufacturer's manual. The qPCR mix (USB VeriQuest SYBR Green qPCR Master Mix 2X, Affymetrix, and primers, according to **Table 4** and **Fig. S3**) was run for 40 cycles in the QuantStudio qPCR machine (Thermo Fisher Scientific) and analyzed with the correspondent software. Differential expression between biocide exposed and control cells was calculated as $\log_2$FC of the relative expression to the *ihfB* gene.

P-value calculation

The p-values, when mentioned, were calculated using t-test (one-tailed) for two independent means.

Comparison between the two sets of controls

To evaluate the quality of the transcriptomic data, we analyzed the differential expression between the pre-inoculum control (cells grown for 12h in M9 glucose without biocide) and the 12h control (cells grown for 12h in M9 glucose without biocide and, after 1:100 dilution in the same media, for additional 7 hours) and compared to the DEGs obtained for biocide exposure. Both sets of controls represented the transcriptomic response of *E.coli* to the media alone (without biocides). The high number of DEGs between the two control groups (235 genes, with 39 of those having absolute $\log_2$FC>1) indicated the occurrence of medium adaptation. Only a few of these DEGs were also present in the biocide's lists of DEGs except for POV30 (44% overlap) and H_2O_2 (43% overlap). As expected, the medium adaptation genes were found to be related to metabolism**.**

Antibiotic resistance

The MIC (minimal inhibitory concentration) of *E. coli* was determined for four antibiotics (vancomycin, chloramphenicol, ampicillin, and novobiocin). For that, cells were grown for 12h in M9 glucose, and 30 µL were added to 3mL tubes containing the biocides specified above, or no biocide (control). After 7 to 12h growth, the OD600 nm was adjusted to approximately 0.05 and 2µL were added to a 96 well plate containing 100 µL of M9 glucose per well, and various concentrations of the antibiotics. Cell concentration at time zero was between 2×10^5 and 8×10^5, as defined by the Clinical and Laboratory Standards Institute (1) and determined by plating samples on LB agar and counting the colonies. The 96 well plates were incubated in the microplate plate reader (Synergy, Biotek) for 24h at 37ºC.

SUPPLEMENTARY RESULTS

Differential Expression Analysis of Short and Long-term Response

We evaluated the gene expression for *Escherichia coli* after continued “short-term” (30 min) and “long-term” (8h-12h) exposure to ten biocides. ETOH, ISOP, and XID treatments resulted in a higher number of DEGs, while exposure to GLUTA resulted in differential expression of 170 genes, most of them with a fold-change lower than two. Both H_2O_2 and BENZ exposure resulted in fewer DEGs compared to other conditions. More than 50 genes had an average log_2FC lower than -3 across biocides, while more than 40 were overexpressed with a log_2FC average above 3 (**Table S1**). Treatments with SOD and ISOP had the most considerable effect on gene expression. For SOD, several DEGs had values of log2FC higher than 5, while the top ten genes mostly downregulated (log2FC <5) were observed for ISOP treatment.

The gene networks for the high-level regulated genes (those with absolute $log_2FC>1$) were represented using Cystoscope. Their clustering demonstrated the enrichment for specific biological processes such as respiration, motility, chemotaxis, protein folding and response to acid (**Fig. S4**).

Differential expression of amino acid biosynthesis and transport and biotin metabolism genes suggested a variation in amino acid requirement after exposure to each biocide.

The levels of expression of amino acid biosynthesis and transport genes were variable depending on the biocide and exposure time. ETOH exposure resulted mainly in the downregulation of genes related to biosynthesis and transport of many amino acids such as arginine, histidine, leucine, valine/ phenylalanine, lysine, and tryptophan. Similarly, exposure to the other alcohol tested, ISOP, resulted in downregulation of comparable groups of genes, as well as additional ones, related to the metabolism of aromatic amino acids. The genes *ilvBN* were upregulated in both alcohols. The two alcohols clustered together regarding their amino acid gene expression (**Fig. S5**). Exposure to PERA, contrastingly, resulted mainly in the overexpression of amino acid related genes. Both arginine and histidine biosynthesis-related genes were upregulated, as well as methionine transport and biosynthesis and isoleucine/valine, after exposure to PERA. Exposure to XID resulted in the higher distance from any other biocide regarding amino acid related gene expression (**Fig. S5**). Exposure to GLUTA resulted in differential expression of several amino acid-related genes, however, in lower levels (lower absolute log_2FC). The expression of amino-acid related genes was distinct between short and long-term exposure to the same biocide, for the biocides tested in both conditions (PHE, POV, and BENZ). The variation of amino acid biosynthesis and transport regulation across different biocides and exposure times could indicate a variable cell necessity for each amino acid (and protein synthesis) for each stress situation. Despite the differences observed across treatments, a few genes related to amino acid biosynthesis were consistently differentially expressed in the same direction. The amino acid dehydrogenase *dadA*, for example, an enzyme in the alanine degradation pathway, was upregulated in ETOH, ISOP, XID ($log_2FC >1$), GLUTA, PERA, PHE, POV and SOD (log_2FC between 0.6 and 1). Such observation is troublesome since alanine catabolism through *dadAX* has been demonstrated to be a preferential route for *Pseudomonas aeruginosa* carbon utilization during host infection. The ability to utilize DL-alanine gave the bacteria a competitive advantage during infection, compared to the *dadAX* knockout mutant (2).

If consistent across genera, the upregulation of such genes after biocide exposure could potentially render bacteria more prepared for subsequent host infection. Genes related to biotin (an essential cofactor for several enzymes biosynthesis) were also upregulated after exposure to several biocides: *bioA* (ETOH, XID), *bioB* (ETOH, ISOP, PHE30, XID), *bioF* (ETOH, XID), *ynfK* (PERA, PHE).

Galactitol metabolism and transport was affected by biocide stress.

Galactitol metabolism and transport were differentially regulated in several conditions (high level: ETOH, ISOP, and XID; low-level: GLUTA and PERA) after long-term exposure of *E. coli* to these biocides. The genes *gatABC*, encoding for the galactitol permease, as well as *gatYZ* (galactitol degradation enzyme) and *gatD* (galactitol dehydrogenase), were upregulated in ETOH, ISOP, and PERA. In contrast, *gatA* and *gatD* were moderately downregulated in XID, and *gatD* was moderately downregulated in GLUTA. The short-term response, contrastingly, tended towards downregulation of this pathway: genes encoding for galactitol metabolism (*gatABCD, gatYZ*) were highly downregulated after exposure to BENZ30, and moderately to POV30 (**Table S1**). GatY showed increased levels in low pH (3) and a mutation in this gene of *E. coli,* combined with additional mutations was associated to increased tolerance to isobutanol (4). Mutations in the *gat* operon were selected for during host infection in the gut by *E. coli* (5), suggesting an importance of such pathway's regulation for bacterial infection.

Glycerol metabolism was altered after exposure to most biocides.

The *glpABC* operon, involved in anaerobic glycerol utilization as a carbon source, was upregulated in PERA, PHE, POV, SOD, and GLUTA treatments. The *glpABC* operon is actively transcribed in anaerobic conditions (6) and has been suggested to play a role in the establishment of persister cells (7). Contrastingly, the *glpABC* operon was downregulated after short-term exposure to biocides (PHE30, BENZ30, POV30), highlighting the difference between the short and long-term response to biocide stress, and the switch into anaerobiosis at a late stage after exposure. The glycerol channel *glpF*, which allows glycerol and additional molecules to cross the membrane towards inside of the cell, was downregulated in most biocides: PERA, SOD, PHE, POV, and XID, as well as short-term exposed POV30 and BENZ30. However, such glycerol transporter was upregulated in ISOP (and to a lesser extent, in ETOH), two alcohols that share a similar structure to glycerol. Knockout mutants for *glpF* were shown to be more resistant to ethanol, tetracycline, DMSO, and stress caused by low osmolarity (8). We observed the same pattern for the glycerol kinase *glpK*, which is part of the same operon and interacts directly with *glpF*. The gene was upregulated in both alcohols: ISOP (and to a lesser extent, in ETOH) but downregulated in XID, POV, and BENZ30 (and to a lesser extent, in PERA, PHE, and SOD). Similarly, the glycerol-3-phosphate transporter *glpT* was upregulated in ISOP and downregulated in GLUTA, PHE, SOD, XID, BENZA30, and POV30. It is possible that ethanol and isopropanol could be inducing the expression of glycerol-related genes due to the molecules' structure similarity to glycerol, which actively binds *glpR*, the repressor of such operons, and further study is required to validate such link.

Exposure to biocides altered gene expression of genes related to transport.

Transport of carbohydrates, peptides and other compounds across the *E. coli* membranes was a functional class differentially regulated in all treatments (**Fig. 3A, Table S1),** even though the genes differentially expressed varied across biocides and the direction of regulation (up or down) was not the same for all genes. Zinc transport trough *znuABC* was highly upregulated for SOD treatment. The genes *znuA* and *znuB* have been shown to be overexpressed in *E. coli* exposed to several different acids (9). Both *znuA* and *znuC* were suggested to participate in a network of genes responsible for DNA repair (10). The ferrous iron transporters *feoAB*, as well as the other member of the operon (*feoC*), were upregulated in multiple conditions: PERA, PHE, POV, SOD, XID, GLUTA, and ETOH. It has been suggested that such iron transporters are important during low oxygen conditions for bacterial iron homeostasis (11). Differential expression of the amino acid transporters and dipeptide transporters was also observed. XID exposure resulted in upregulation of the peptide transporter *yjiY*. This gene was recently shown to be involved in infection and pathogenesis of Salmonella (12). Phosphate ABC transporters *pstA*, *pstC*, and *pstS* were upregulated in both ETOH and ISOP. The *pstS* transporter has been linked to antibiotic resistance for *Streptococcus pneumoniae*, in which higher levels of mRNA for this gene correlated to higher resistance to penicillin in clinical isolates (13).

Enterobactin biosynthesis was downregulated after short-term exposure to chlorophene.

Genes encoding for the iron-chelating enterobactin biosynthesis pathway, *entCEBA,* and *entF,* were downregulated in cells exposed to PHE for 30 minutes ($log_2FC<1$), which was not observed for long-term exposure to the same biocide (**Table S1**). Lower levels of differential expression of a few enterobactin biosynthesis genes were also observed for cells exposed to other biocides, such as ISOP. Recently, enterobactin was shown to have fundamental importance in protection against oxidative stress (14, 15), besides its well-known role as an iron acquirer (siderophore). While upregulation of enterobactin-related genes could be explained by the oxidative stress response caused by a biocide, it is unclear for us what could be the downstream result of downregulation after exposure to PHE30.

SUPPLEMENTARY REFERENCES

1. Clinical and Laboratory Standards Institute CLSI. 2012. Methods for Dilution Antimicrobial Susceptibility Tests for Bacteria That Grow Aerobically; Approved Standard. 9 32.

2. Boulette ML, Baynham PJ, Jorth PA, Kukavica-Ibrulj I, Longoria A, Barrera K, Levesque RC, Whiteley M. 2009. Characterization of alanine catabolism in Pseudomonas aeruginosa and its importance for proliferation in vivo. J Bacteriol 191:6329–6334.

3. Yohannes E, Barnhart DM, Slonczewski JL. 2004. pH-dependent catabolic protein expression during anaerobic growth of Escherichia coli K-12. J Bacteriol 186:192–199.

4. Atsumi S, Wu T-Y, Machado IM, Huang W-C, Chen P-Y, Pellegrini M, Liao JC. 2010. Evolution, genomic analysis, and reconstruction of isobutanol tolerance in Escherichia coli. Mol Syst Biol 6.

5. Barroso-Batista J, Sousa A, Lourenço M, Bergman M-L, Sobral D, Demengeot J, Xavier KB, Gordo I. 2014. The first steps of adaptation of Escherichia coli to the gut are dominated by soft sweeps. PLoS Genet 10.

6. Cole S, Eiglmeier K, Ahmed S, Honore N, Elmes L, Anderson W, Weiner J. 1988. Nucleotide sequence and gene-polypeptide relationships of the glpABC operon encoding the anaerobic sn-glycerol-3-phosphate dehydrogenase of Escherichia coli K-12. J Bacteriol 170:2448–2456.

7. Spoering AL, Vulić M, Lewis K. 2006. GlpD and PlsB participate in persister cell formation in Escherichia coli. J Bacteriol 188:5136–5144.

8. Truniger V, Boos W. 1993. Glycerol uptake in Escherichia coli is sensitive to membrane lipid composition. Res Microbiol 144:565–574.

9. King T, Lucchini S, Hinton JC, Gobius K. 2010. Transcriptomic analysis of Escherichia coli O157: H7 and K-12 cultures exposed to inorganic and organic acids in stationary phase reveals acidulant-and strain-specific acid tolerance responses. Appl Env Microbiol 76:6514–6528.

10. Al Mamun AAM, Lombardo M-J, Shee C, Lisewski AM, Gonzalez C, Lin D, Nehring RB, Saint-Ruf C, Gibson JL, Frisch RL, others. 2012. Identity and function of a large gene network underlying mutagenic repair of DNA breaks. Science 338:1344–1348.

11. Andrews SC, Robinson AK, Rodríguez-Quiñones F. 2003. Bacterial iron homeostasis. FEMS Microbiol Rev 27:215–237.

12. Garai P, Chandra K, Chakravortty D. 2017. Bacterial peptide transporters: Messengers of nutrition to virulence. Virulence 8:297–309.

13. Soualhine H, Brochu V, Ménard F, Papadopoulou B, Weiss K, Bergeron MG, Légaré D, Drummelsmith J, Ouellette M. 2005. A proteomic analysis of penicillin resistance in Streptococcus pneumoniae reveals a novel role for PstS, a subunit of the phosphate ABC transporter. Mol Microbiol 58:1430–1440.

14. Adler C, Corbalan NS, Peralta DR, Pomares MF, de Cristóbal RE, Vincent PA. 2014. The alternative role of enterobactin as an oxidative stress protector allows Escherichia coli colony development. PloS One 9.

15. Peralta DR, Adler C, Corbalán NS, García ECP, Pomares MF, Vincent PA. 2016. Enterobactin as part of the oxidative stress response repertoire. PloS One 11.

16. Baba T, Ara T, Hasegawa M, Takai Y, Okumura Y, Baba M, Datsenko KA, Tomita M, Wanner BL, Mori H. 2006. Construction of Escherichia coli K-12 in-frame, single-gene knockout mutants: the Keio collection. Mol Syst Biol 2.

SUPPLEMENTARY TABLES

Table S1. (Excel file). Complete list of DEGs (differentially expressed genes) and GO (gene ontology) terms for the DEGs that are both in Deseq and EdgeR methods.

Table S2

Condition	Total number of DEGs			
	Dseq	EdgeR	Shared (in both Dseq and EdgeR)	with $log_2FC<-1$ or >1
BENZ	57	39	35	35
ETOH	980	780	598	101
GLUTA	323	280	170	26
H2O2	25	65	14	6
ISOP	1174	823	672	179
PERA	589	384	296	129
PHE	840	545	421	113
POV	397	242	204	134
SOD	1055	610	488	98
XID	1143	681	585	201
BENZ30	669	530	396	106
PHE30	596	484	341	91
POV30	368	301	205	35
CONTROL_control	425	391	235	39
average	632	443	340	96

Table S2. Total number of differentially expressed genes (DEGs) according to Dseq and EdgeR (DEGs were defined as those with qvalue <=0.05), DEGs shared among both methods and with log_2FoldChange <-1 or >1.

Table S3

Condition	Top upregulated			Top downregulated		
	**Gene**	**log_2FC**	**Biological process**	**Gene**	**log_2FC**	**Biological process**
BENZ	N/A	N/A	N/A	*crfC*	-1.90	DNA replication
ETOH	*asr*	5.26	response to acid	*puuA*	-4.18	putrescine catabolic process
GLUTA	*cueO*	2.02	oxidation-reduction; response to copper	*dinQ*	-1.64	response to DNA damage
H_2O_2	*mntH*	0.74	ion transport	*sdhC*	-1.38	TCA cycle
ISOP	*asr*	4.76	response to acid	*fliC*	-5.92	flagellum-dependent cell motility
PERA	*znuA*	5.26	zinc and ion transport	*puuA*	-3.14	putrescine catabolic process
PHE	*bssR*	4.00	biofilm regulation	*puuA*	-3.04	putrescine catabolic process
POV	*hdeA*	3.99	response to acid; response to stress	*puuA*	-2.68	putrescine catabolic process
SOD	*zinT*	8.74	response to cadmium; response to zinc starvation	*puuA*	-3.52	putrescine catabolic process
XID	*yjiY*	4.51	transport; response to starvation	*fliK*	-4.07	flagellum-dependent cell motility
BENZ30	*cpxP*	2.61	response to stress; regulation of proteolysis	*glpT*	-2.30	transport; glycerol metabolic process
PHE30	*sodB*	3.27	response to superoxide	*yjjZ*	-3.75	putative protein
POV30	*ibpB*	6.11	response to heat; response to copper	*uraA*	-2.41	uracil transmembrane transport
Controls	*uraA*	1.80	uracil transmembrane transport	*stpA*	-1.73	regulation of transcription

Table S3. Top up and down-regulated genes (with highest absolute log_2 Fold Change) for each condition, with corresponding log_2FC and biological process. N/A: not applicable

SUPPLEMENTARY FIGURES LEGENDS

Fig. S1. **A**. The average between the absolute log_2FC of DEGs belonging to the same category was calculated, and the biological processes were organized from the most differentially regulated to the least differentially regulated, for the majority of the biocides. A darker color represents a process that was more affected by the indicated biocide. **B**. PCA (principal component analysis) for DEGs using EdgeR. **C**. Weights for the 10 DEGs with the highest weights for PCA1 and PCA2 of Fig. 3B. **D**. Weights for the 10 DEGs with the highest weights for PCA1 and PCA2 of Fig. S1B.

Fig S2. Representation of genomic areas amplified from Keio knockout strains for sequence check and validation of the correct insertion of kanamycin cassette as described in (16). The sequencing primers were design to align within the kanamycin gene, as indicated.

Fig. S3. Comparison between qPCR and RNAseq data for selected genes. Log_2FC average of at least two biological replicates. Error bars represent standard error for qPCR and qvalues for RNAseq.

Fig. S4. Gene network for *E. coli* exposed to biocides. DEGs with absolute log2FC higher than one were organized into networks with Cytoscape and STRING (default settings). Thicker lines between nodes indicate higher confidence for the gene interaction. Arrows indicate clusters which contain DEGs that had high levels of regulation after biocide exposure: green- chaperones, pink- enterobactin, black- motility, and chemotaxis, blue- respiration, red- amino acids, orange- response to acid.

Fig. S5. Dendrogram for amino acid related genes. All DEGs related to amino acid transport or biosynthesis (based on GO terms) for each biocide were included. Euclidean distance and Ward clustering were used.

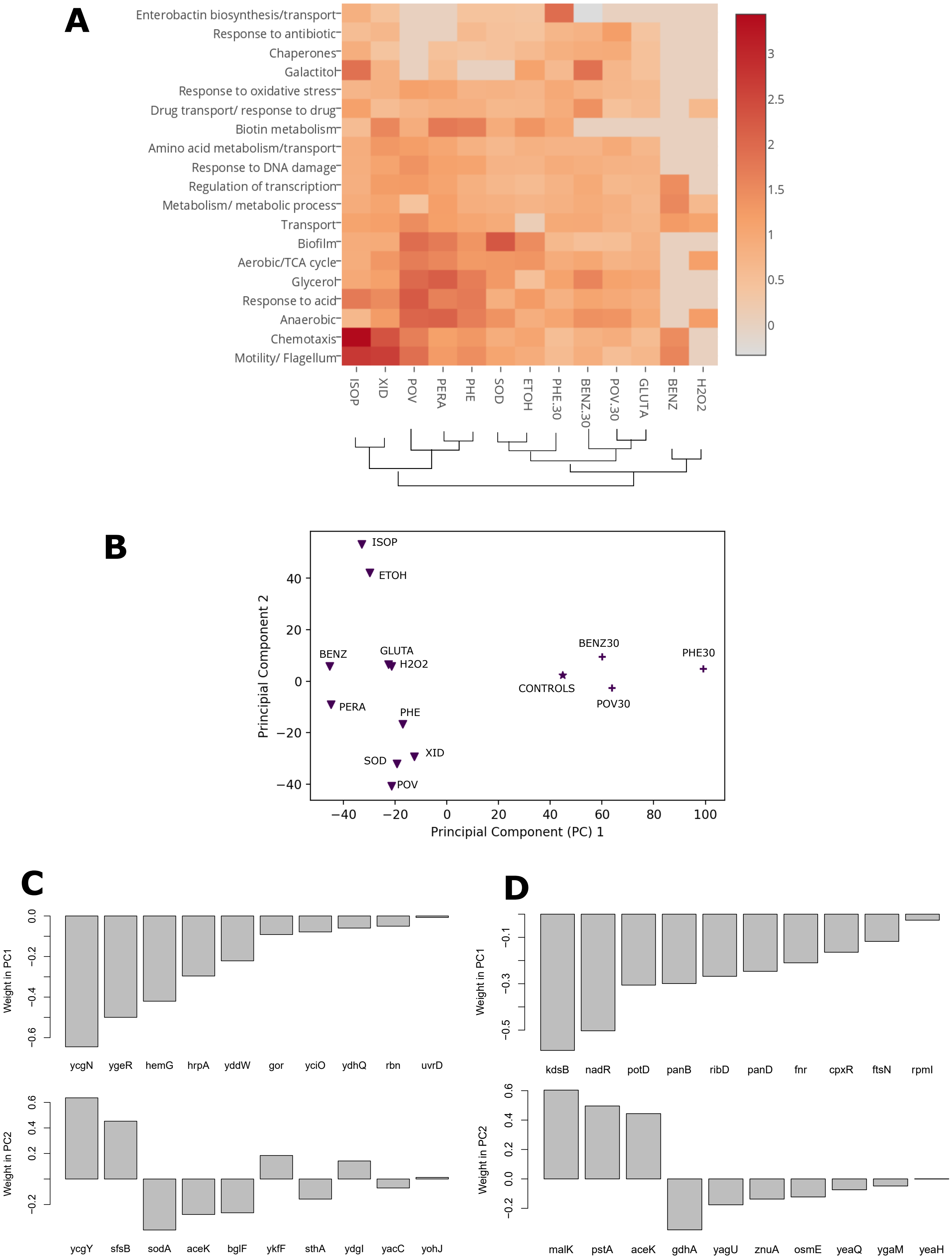

A
Enterobactin biosynthesis/transport
Response to antibiotic
Chaperones
Galactitol
Response to oxidative stress
Drug transport/ response to drug
Biotin metabolism
Amino acid metabolism/transport
Response to DNA damage
Regulation of transcription
Metabolism/ metabolic process
Transport
Biofilm
Aerobic/TCA cycle
Glycerol
Response to acid
Anaerobic
Chemotaxis
Motility/ Flagellum
ISOP
XID
POV
PERA
PHE
SOD
ETOH
PHE.30
BENZ.30
POV.30
GLUTA
BENZ
H2O2
3
2.5
2
1.5
1
0.5
0
B
ISOP
ETOH
BENZ
GLUTA
H2O2
BENZ30
PHE30
CONTROLS
POV30
PERA
PHE
SOD
XID
POV
Principial Component 2
Principial Component (PC) 1
C
Weight in PC1
ycgN
ygeR
hemG
hrpA
yddW
gor
yciO
ydhQ
rbn
uvrD
Weight in PC2
ycgY
sfsB
sodA
aceK
bglF
ykfF
sthA
ydgI
yacC
yohJ
D
Weight in PC1
kdsB
nadR
potD
panB
ribD
panD
fnr
cpxR
ftsN
rpmI
Weight in PC2
malK
pstA
aceK
gdhA
yagU
znuA
osmE
yeaQ
ygaM
yeaH

Fig. S2

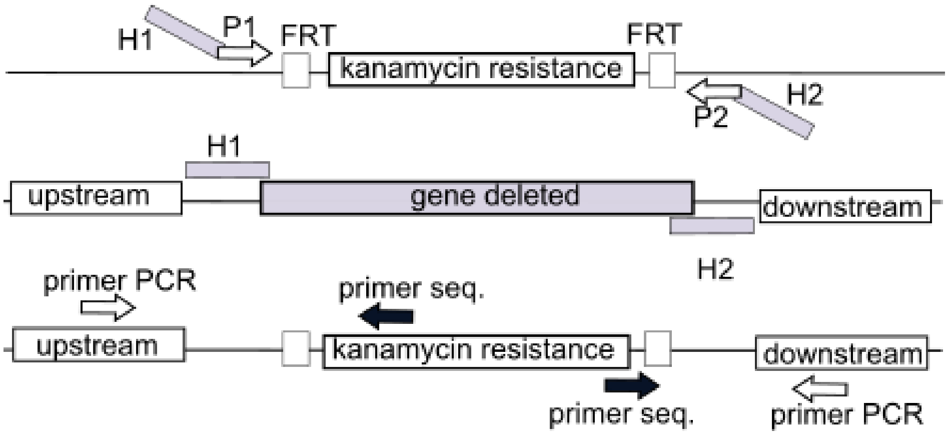
H1
P1
FRT
kanamycin resistance
FRT
H2
P2
H1
upstream
gene deleted
downstream
H2
primer PCR
primer seq.
upstream
kanamycin resistance
downstream
primer seq.
primer PCR

Fig S3

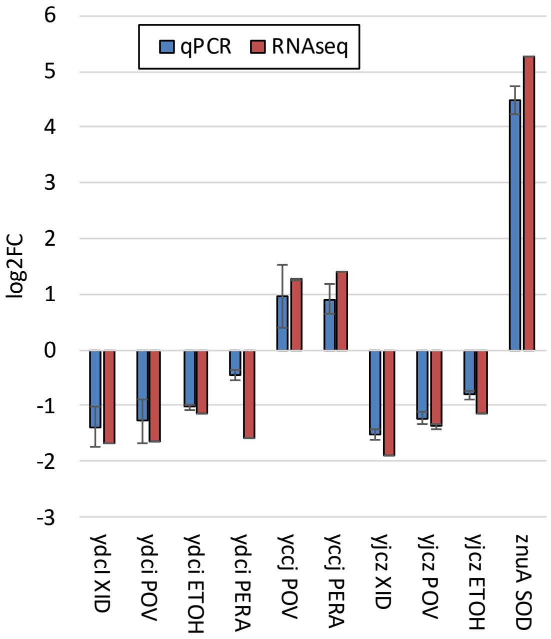
qPCR
RNAseq
log2FC
6
5
4
3
2
1
0
-1
-2
-3
ydcI XID
ydci POV
ydci ETOH
ydci PERA
yccj POV
yccj PERA
yjcz XID
yjcz POV
yjcz ETOH
znuA SOD

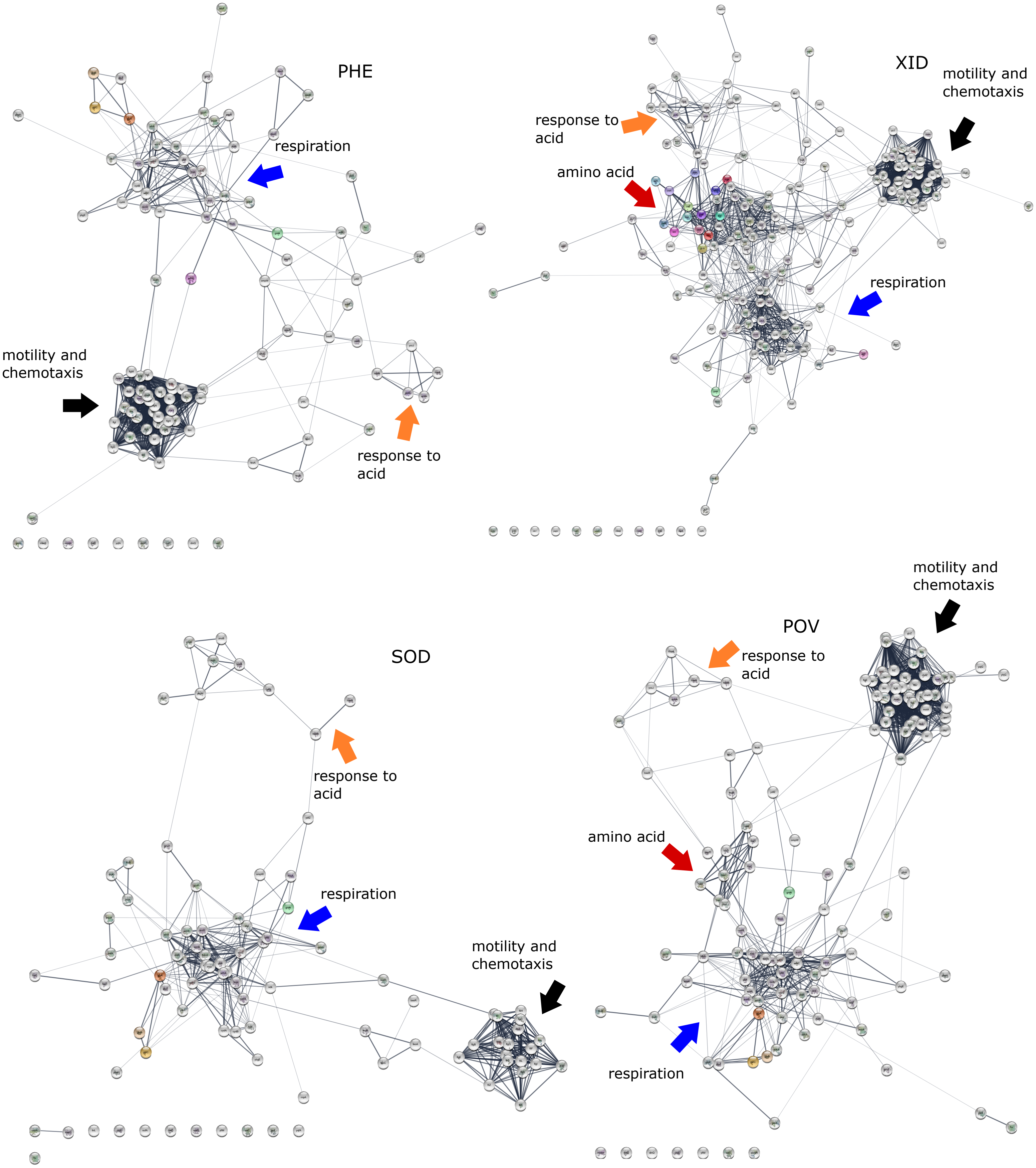
PHE
respiration
motility and chemotaxis
response to acid
XID
motility and chemotaxis
response to acid
amino acid
respiration
SOD
response to acid
respiration
motility and chemotaxis
POV
motility and chemotaxis
response to acid
amino acid
respiration

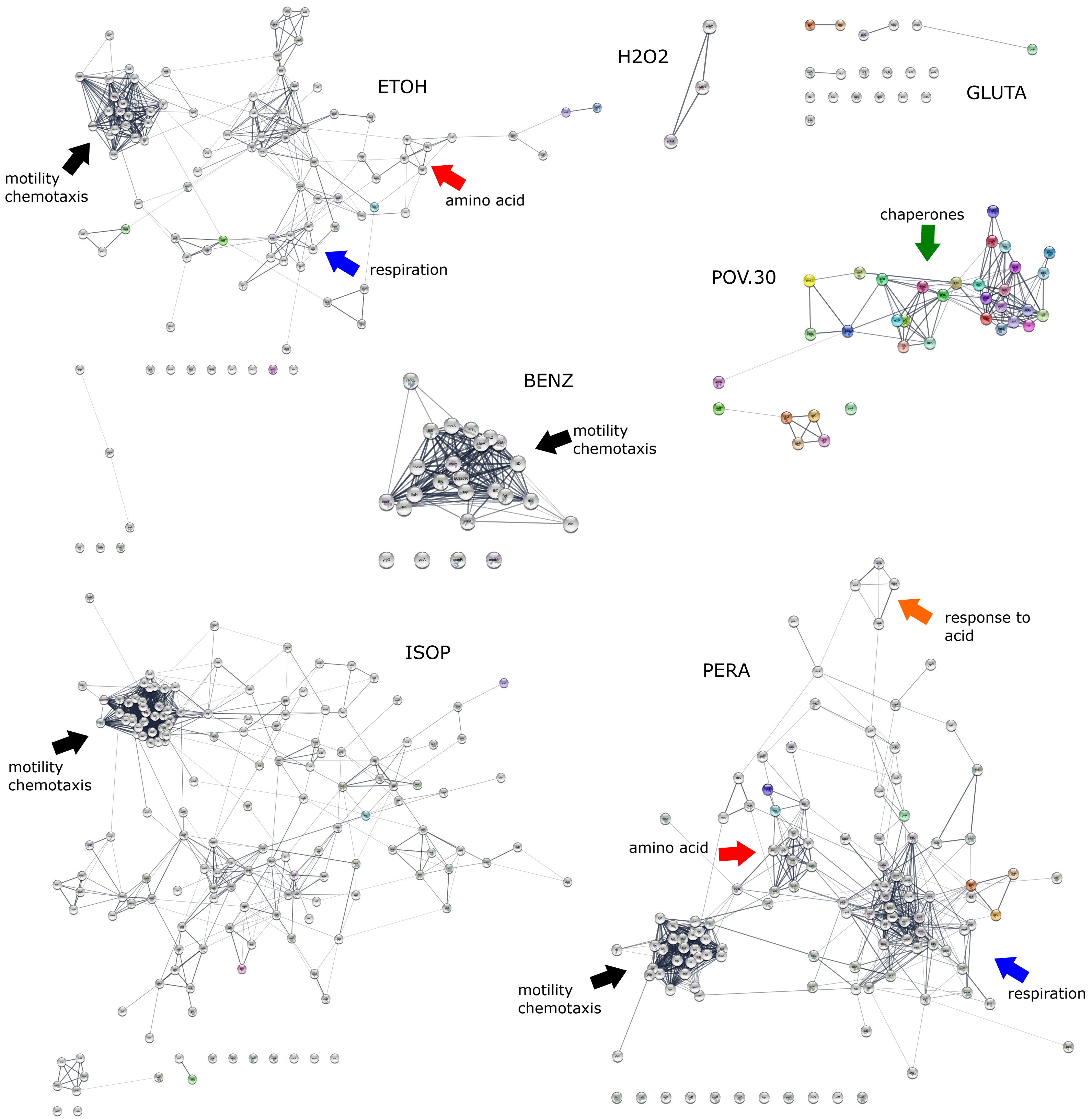

ETOH
motility
chemotaxis
amino acid
respiration
H2O2
GLUTA
chaperones
POV.30
BENZ
motility
chemotaxis
ISOP
motility
chemotaxis
response to
acid
PERA
amino acid
motility
chemotaxis
respiration

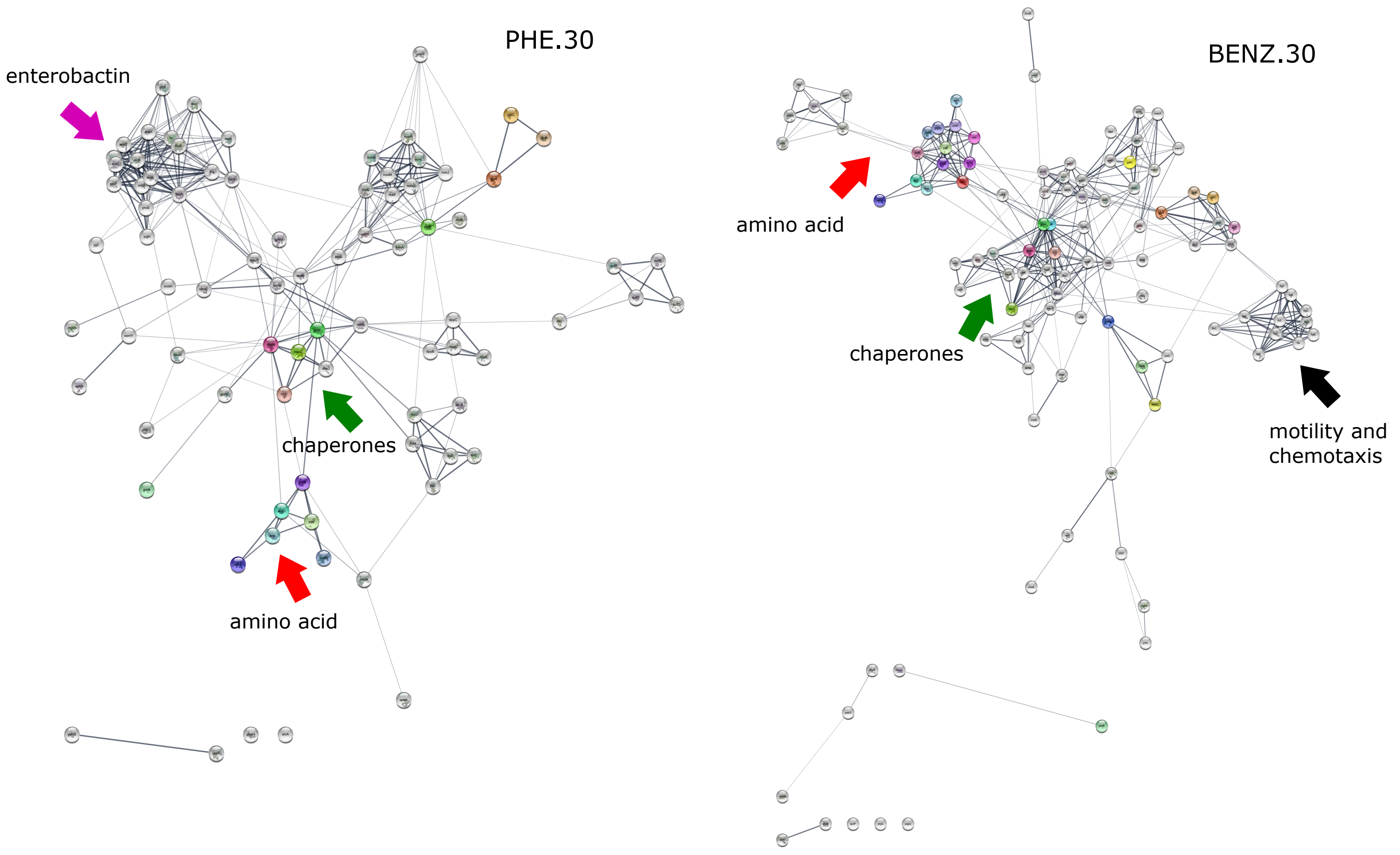
PHE.30
enterobactin
chaperones
amino acid
BENZ.30
amino acid
chaperones
motility and
chemotaxis

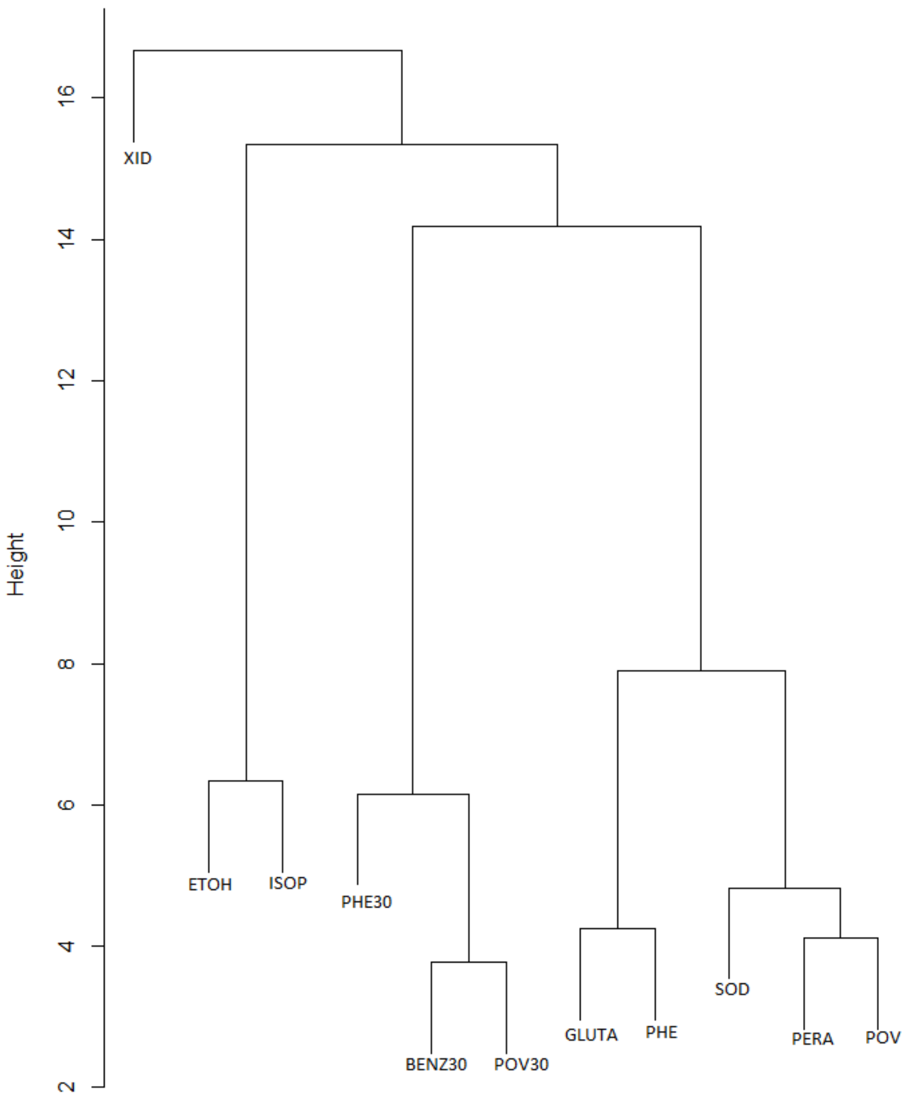
Height
16
14
12
10
8
6
4
2
XID
ETOH
ISOP
PHE30
BENZ30
POV30
GLUTA
PHE
SOD
PERA
POV
